# Performance of fasting plasma glucose for community-based screening of undiagnosed diabetes and pre-diabetes in sub-Saharan Africa

**DOI:** 10.3389/fendo.2025.1501383

**Published:** 2025-03-25

**Authors:** Assefa Mulu Baye, Teferi Gedif Fenta, Suvi Karuranga, Ifeyinwa Dorothy Nnakenyi, Ekenechukwu Esther Young, Colin Palmer, Ewan R. Pearson, Ifeoma Isabella Ulasi, Adem Y. Dawed

**Affiliations:** ^1^ Department of Pharmacology and Clinical Pharmacy, College of Health Sciences, Addis Ababa University, Addis Ababa, Ethiopia; ^2^ Department of Pharmaceutics and Social Pharmacy, College of Health Sciences, Addis Ababa University, Addis Ababa, Ethiopia; ^3^ European Society for Emergency Medicine, Antwerp, Belgium; ^4^ Department of Chemical Pathology, College of Medicine, University of Nigeria & University of Nigeria Teaching Hospital, Enugu, Nigeria; ^5^ Department of Medicine, College of Medicine, University of Nigeria & University of Nigeria Teaching Hospital, Ituku-Ozalla, Enugu, Nigeria; ^6^ Division of Population Health and Genomics, Ninewells Hospital and School of Medicine, University of Dundee, Dundee, United Kingdom; ^7^ Department of Internal Medicine, Alex Ekwueme Federal University Teaching Hospital, Abakaliki, Nigeria

**Keywords:** diabetes, fasting plasma glucose, 2-h plasma glucose, sub-Saharan Africa, sensitivity, specificity

## Abstract

**Introduction:**

Early diabetes screening is critical in sub-Saharan Africa (SSA), where the prevalence is increasing, yet a large proportion of cases remain undiagnosed. This study aimed to evaluate the performance of fasting plasma glucose (FPG) in screening diabetes and/or prediabetes compared to the 2-hour plasma glucose (2-h PG)-level in SSA.

**Methods:**

Data from a population-based, cross-sectional diabetes screening survey involving 1550 individuals in Butajira, Ethiopia, and Enugu state, Nigeria were analyzed. Fasting plasma glucose and a 2-hour 75-g oral glucose tolerance test (OGTT) were utilized for diabetes screening. In addition, we determined and plotted the receiver operating characteristic curve for FPG against the reference standard 2-h PG to evaluate the screening tool’s sensitivity and specificity.

**Results:**

The mean (SD) age of the study participants was 44.5 (± 16.43) years, with men comprising 50.4% of the cohort. Among 1550 individuals analyzed, 4.6% and 16.8% demonstrated diabetes and prediabetes, respectively, as identified by either FPG or 2-h PG. The agreement between FPG and 2-h PG in identifying diabetes and prediabetes was moderate, with kappa statistic of 0.56 (95% CI, 0.51 – 0.61; p<0.0001) for diabetes and 0.45 (95% CI, 0.40 – 0.50; p<0.0001) for prediabetes. FPG failed to detect 34.1% of all prediabetes and 44.4% of all diabetes cases. The sensitivity of FPG in identifying diabetes cases was 44.3% at a cut-off 126 mg/dL with a specificity of 99.3%. We identified the optimal FPG cut-off for detecting newly identified diabetes cases using 2-h PG to be 105 mg/dL associated with a sensitivity and specificity of 67.2% and 94.0%, respectively.

**Conclusion:**

FPG was able to correctly identify 99.3% of individuals with no diabetes but a significant percentage of diabetes cases would have remained undiagnosed if only FPG had been utilized instead of the 2-h PG. The use of 2-h PG test is recommended to diagnose diabetes in older individuals, females and non-obese persons who would be missed if tested by only FPG. Lowering the cut-off value for FPG to 105 mg/dL substantially increases the identification of individuals with diabetes, thus improving the effectiveness of FPG as a screening test for type 2 diabetes.

## 1 Introduction

Diabetes is an important worldwide health concern, with 830 cases (14% of the adult population aged 18 years and older) were living with diabetes (1) and 6.7 million deaths among adults in 2021 (2). Projections from the International Diabetes Federation (IDF) forecast a surge to 643 million cases by 2030. In addition, diabetes results in serious complications such as visual impairment and blindness, kidney problems, heart diseases, and cerebrovascular diseases (3).

Societal guidelines stressed the pivotal role of early detection of type 2 diabetes to ensure effective glycemic control and delay the onset of complications (4–6). Community-based screening for type 2 diabetes is important for early detection of the disease as it is asymptomatic and high rates of undiagnosed cases (7).

The American Diabetes Association (ADA) and IDF recommend FPG and the 2-hour postprandial oral glucose tolerance test (OGTT) for the diagnosis of prediabetes and diabetes (4, 8). Each method has distinct advantages and disadvantages in clinical application. Diagnosis of diabetes by FPG is preferred owing to its cost-effectiveness and convenience, particularly in identifying high risk individuals with diabetes. However, the 2-hour postprandial glucose (2-h PG) test shows superior sensitivity for identifying individuals with prediabetes and diabetes, although being less feasible in large-scale mass screening (8). As a result, 2-h PG is considered to be the gold standard in the diagnosis of diabetes (4).

According to ADA, the criteria for the diagnosis of diabetes are FPG ≥126 mg/dL and/or 2-h PG ≥200 mg/dL (using a glucose load containing the equivalent of 75 g anhydrous glucose dissolved in water) and/or A1C ≥6.5% and/or a random plasma glucose ≥200 mg/dL in a person with classic symptoms of hyperglycemia or hyperglycemic complications (4). There is an ever-growing discourse underway regarding the current thresholds suggested for the diagnosis and screening of diabetes and prediabetes (9, 10). The establishment of these threshold values primarily relied on the outcomes of several studies conducted in Western populations, aiming to identify the most effective FPG and other screening tests’ cut-off values for predicting diabetes (11). Several cohort studies among Asian populations examined FPG and recommended lower cut-off values to predict diabetes (12–15). However, epidemiological studies which investigated comparative performance and cut-off values of FPG for predicting type 2 diabetes in Sub-Saharan Africa (SSA) population are scarce and it remains uncertain whether a FPG value of 126 mg/dL is adequate in screening type 2 diabetes in this region. In addition, evidence is limited in investigating those individuals whose diabetes status could be classified differently by different screening tests. Such lack of agreement may stem from measurement inconsistencies, variation with time, or screening tests measure their respective physiological processes (16). ADA, IDF and the European Association for the Study of Diabetes (EASD) also indicated that these blood-based methodologies have limitations in interpretation variabilities and missing cases related to concomitant conditions such as haemoglobinopathies and anemias (4, 8, 17). This is particularly critical in SSA, where the high prevalence of such comorbidities may contribute to missed cases or variability in interpretations of test results.

Few epidemiological investigations in SSA, especially across Ethiopia and Nigeria, have directly assessed the utility of FPG in identifying undiagnosed type 2 diabetes and prediabetes individuals. Therefore, this research aims to elucidate the screening capacity (sensitivity and specificity) and determine optimal cut-offs for FPG based on 2-h PG-detected prediabetes and diabetes. The outcomes will offer significant insights for healthcare providers, managers and policymakers involved in diabetes and prediabetes care in SSA.

## 2 Materials and methods

### 2.1 Study design and setting

The Diabetes Epidemiologists’ Network in Sub-Saharan Africa (DENSSA): represents collaborative research initiative involving the University of Dundee in United Kingdom, Addis Ababa University in Ethiopia and University of Nigeria in Nigeria. Within this framework, a community-based, cross-sectional study was conducted as a crucial facet of the project, encompassing the Butajira Rural Health Program (BRHP) in central Ethiopia and at Enugu State in Nigeria. Data was collected between November 2020 to October, 2021.

### 2.2 Study participants

The study participants comprised individuals from six villages in Butajira, Ethiopia and three Local Government Areas (LGAs) – Enugu North, Enugu West and Nkanu West in Enugu State, Nigeria. Utilizing a multi-stage random cluster sampling technique, a representative sample of 1000 community-based adults ≥18 years were planned to be selected from each of the two study settings. The sample of participants in each district was allocated using a probability proportional to the size technique. We utilized computer generated list from Butajira Rural Health Program (BRHP) register in Ethiopia and Enugu State in Nigeria to select households. Pregnant women, acutely ill individuals, and those taking medications including steroids, second generation antipsychotics, and protease inhibitors as well as those refused to participate were excluded from the study.

### 2.3 Data collection

After study participants were interviewed on the first day, they were requested to visit health centers on the subsequent day, between 7:30 a.m. and 9:00 a.m. following an overnight fast of 10–12 hours. A structured and pre-coded questionnaire administered by a trained interviewer, was used for data collection. Measurement of blood pressure (BP), heart rate, weight, height, waist, and hip circumference were conducted at the nearby health facilities. Blood samples for FPG were collected from participants who had fasted overnight for at least ten hours. Thereafter, all participants drank a standard solution of 75g of anhydrous glucose dissolved in 300ml of water. Two hours after ingesting the drink, a second venous blood sample was collected. The blood samples for glucose estimation were collected into sterilized disposable vacutainer tubes containing sodium fluoride, which were quickly centrifuged within an hour at the data collection center to separate the plasma. Blood samples were stored in cold boxes maintained at 4–8°C until transported to a laboratory on the same day as sample collection. Biochemical tests were conducted on the same day by the International Clinical Laboratories (ICL) for the Ethiopian samples and at the Chemical Pathology laboratory of the University of Nigeria Teaching Hospital for the Nigerian samples to determine blood glucose concentration.

### 2.4 Diagnostic criteria

The ADA’s and IDF’s diagnostic definitions were utilized to identify diabetes and pre-diabetes cases (4, 18). Plasma glucose test results were categorized as follows: impaired fasting glucose (IFG) corresponding to plasma glucose levels between 100 mg/dL and 125 mg/dL, and impaired glucose tolerance (IGT) defined as a 2-h PG ranging from 140 mg/dL and 199 mg/dL. Newly diagnosed diabetes was ascribed to a FPG level of ≥126mg/dL, 2-h PG of ≥200mg/dL or both.

Body mass index (BMI) was delineated according to the WHO criteria into: underweight (< 18.5 kg/m2), normal weight (18.5–24.9 kg/m^2^), overweight (25.0–29.9 kg/m^2^) and obesity (≥ 30 kg/m^2^) (19). Waist circumference was classified as normal (≤ 94 cm for men and ≤ 80 cm for women), presenting an increased risk of cardiometabolic complications with measurements of 95–102 cm for men and 81–88 cm for women, and substantially increased risk of cardiometabolic complications if the waist circumference exceeded > 102 cm for men and > 88 cm women) (20).

Participants’ physical activity was evaluated based on adherence to WHO recommendations, categorizing individuals as either optimal (≥ 600 Metabolic Equivalent of Task (MET) minutes per week) or suboptimal (< 600 MET minutes per week) (21).

### 2.5 Data analysis

We utilized Stata V.17 software program (StataCorp. Stata Statistical Software: Release 17. College Station, TX: Stata Corp LP) and R version 4.3.2, (22) for data analysis. Variables with continuous outcome were reported as mean ± standard deviation (SD) or median with interquartile range (IQR). Differences in means were assessed using an independent Student’s *t*-test. Categorical differences were expressed in proportions and assessed using a Chi-square test. The medians of FPG and 2-h PG were compared across categorical variables utilizing the Wilcoxon rank-sum test or Kruskal-Wallis rank-test. Regression analysis was utilized to evaluate the relationship between FPG and 2-h PG.

The agreement between FPG and 2-h PG was determined using kappa statistics, accompanied by the 95% confidence interval (95% CI). We considered *P* < 0.05 to declare statistically significant. Receiver operating characteristic (ROC) curves were computed and plotted, with the FPG compared to the reference standard 2-h PG to ascertain its diagnostic capacity as a diabetes and prediabetes screening tool. Evaluation metrics such as sensitivity, specificity, and Youden’s Index were employed to assess the performance of FPG tests (23). Youden’s Index, a metric used to express the discriminatory capability of diagnostic tests, is derived from the formula: (sensitivity + specificity) − 1. The index ranges from −1 to 1, with a value closer to 1, indicating superior test performance.

Furthermore, the proportion of missed prediabetes by FPG and 2-h PG, as well as the proportion of missed diabetes cases by FPG, were calculated against the total number of prediabetes and diabetes cases, respectively.

## 3 Results

### 3.1 Sociodemographic and clinical information

Among the total of 1727 participants, 1550 individuals were included in the analysis following the exclusion of 30 previously diagnosed with type 2 diabetes and 147 who were not tested by either FPG or 2-h PG. The mean age of the study population was 44.5 (SD 16.4) years, with women comprising 49.6% of the analyzed cohort (**Table 1**).

**Table 1:** Summary of sociodemographic and clinical information by country of residence, Sub-Sahara Africa, 2021.

Characteristics	Country		Total
	Ethiopia	Nigeria	
Sex, women	394 (63.7)	375 (40.3)	769 (49.6)
Age (years)	40.8 ± 13.5	46.9 ± 17.7	44.5 ± 16.4
Urban residence	354 (57.2)	431 (46.3)	785 (50.60)
BMI (kg/m^2^)^a^	23.3 ± 4.1	28.1 ± 10.3	28.2 ± 8.7
Waist circumference (cm)	76.8 ± 11.0	88.7 ± 14.0	83.9 ± 14.1
WHR^b^	0.86 ± 0.1	–	0.86 ± 0.1
Diastolic BP (mm Hg)	78.9 ± 11.2	81.8 ± 13.1	80.6 ± 12.5
Systolic BP (mm Hg)	119.1 ± 16.7	132.1 ± 21.6	126.9 ± 20.8
FPG (mg/dL)	79 (73-85)	87 (79-96)	83 (76-92)
2-h PG (mg/dL)	81 (67-98)	103 (86-129)	94 (77-116)
IFG	27 (4.4)	145 (15.5)	172 (11.1)
IGT	27 (4.4)	151(16.2)	178 (11.5)
Prediabetes	40 (6.5)	221 (23.7)	261 (16.8)
New diabetes by FPG	4 (0.6)	36 (3.9)	40 (2.6)
New diabetes by 2-h PG	18 (2.9)	43 (4.6)	61 (3.9)
Overall New diabetes	18 (2.9)	54 (5.8)	72 (4.6)
Normal glucose tolerance	561 (90.6)	656 (70.5)	1217 (78.5)

Data are presented as n (%), mean ± SD or median (IQR).

a Seven values were missing.

b WHR is available for the Ethiopian data only.

BMI, body mass index; FPG, fasting plasma glucose; IQR, interquartile range; 2-h PG, 2-hour plasma glucose; SD, standard deviation; WHR, waist-to-hip ratio; IFG, impaired fasting glucose; IGT, impaired glucose tolerance.

Of the 1550 participants, 72 individuals (4.6%) were identified as having diabetes through either FPG or 2-h PG, whereas 261 individuals (16.8%) demonstrated prediabetes, and 1217 individuals (78.5%) exhibited normal glucose tolerance. Among all tested individuals, 11 (15.3%) displayed abnormalities in FPG alone (false positive results), 32 (44.4%) in 2-h PG alone (false negative results for FPG), and 29 (40.3%) in both. Of those with prediabetes, 83 (31.8%) presented with IFG, 89 (34.1%) had IGT and 89 (34.1%) with a combination of both.

### 3.2 Patterns of FPG and 2-h PG results

**Figure 1:**
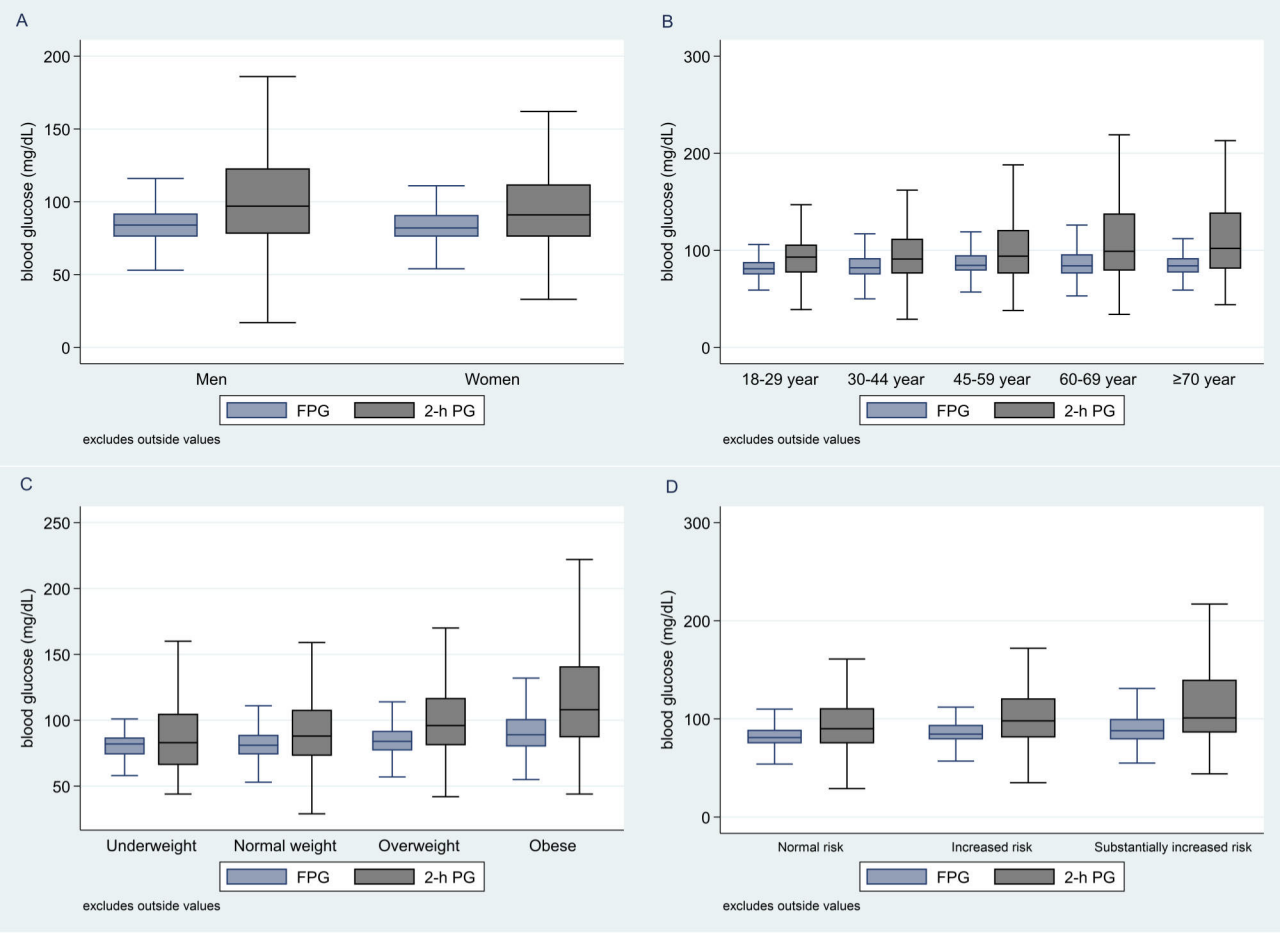
Box plots for median FPG and 2-h PG by sex **(A)**, age category **(B)**, body mass index (BMI) **(C)** and waist circumference **(D)**. FPG, fasting plasma glucose; 2-h PG, 2-hour plasma glucose.

Median FPG among all participants was 83 mg/dL (IQR: 76 – 92), while the median 2-h PG level was 94 mg/dL (IQR: 77-116). Notably, men demonstrated a significantly higher median 2-h PG compared to women (97 mg/dL, IQR: 78-123 vs 91 mg/dL, IQR: 76-112; P < 0.0006) (**Figure 1A**). Moreover, the age group of 45-59 years exhibited the highest median FPG levels (84.5 mg/dL, IQR 79 – 95, p<0.0001), while the ≥70 age group had the highest median 2-h PG levels (102 mg/dL, IQR 81 - 139, p<0.0001) (**Figure 1B**).

In addition, FPG values increased from underweight individuals to those classified as obesity (underweight 82 mg/dL, IQR 74-87; normal weight 81 mg/dL, IQR 74-89; overweight 84 mg/dL, IQR 77-92; obese 89 mg/dL, IQR 80-101; P < 0.0001; as determined by WHO BMI criteria). Likewise, 2-h PG values progressively increased from individual with underweight to obese participants (underweight 83 mg/dL, IQR 66-105; normal weight 88 mg/dL, IQR 73-108; overweight 96 mg/dL, IQR 81-117; obese 108 mg/dL, IQR 87-141; P < 0.0001; by WHO BMI criteria) (**Figure 1C**). Similarly, both FPG and 2hPG showed progressive elevation across low-risk to substantially increased risk groups, based on waist circumference (**Figure 1D**).

### 3.3 Concordance between FPG and 2-h PG

**Figure 2:**
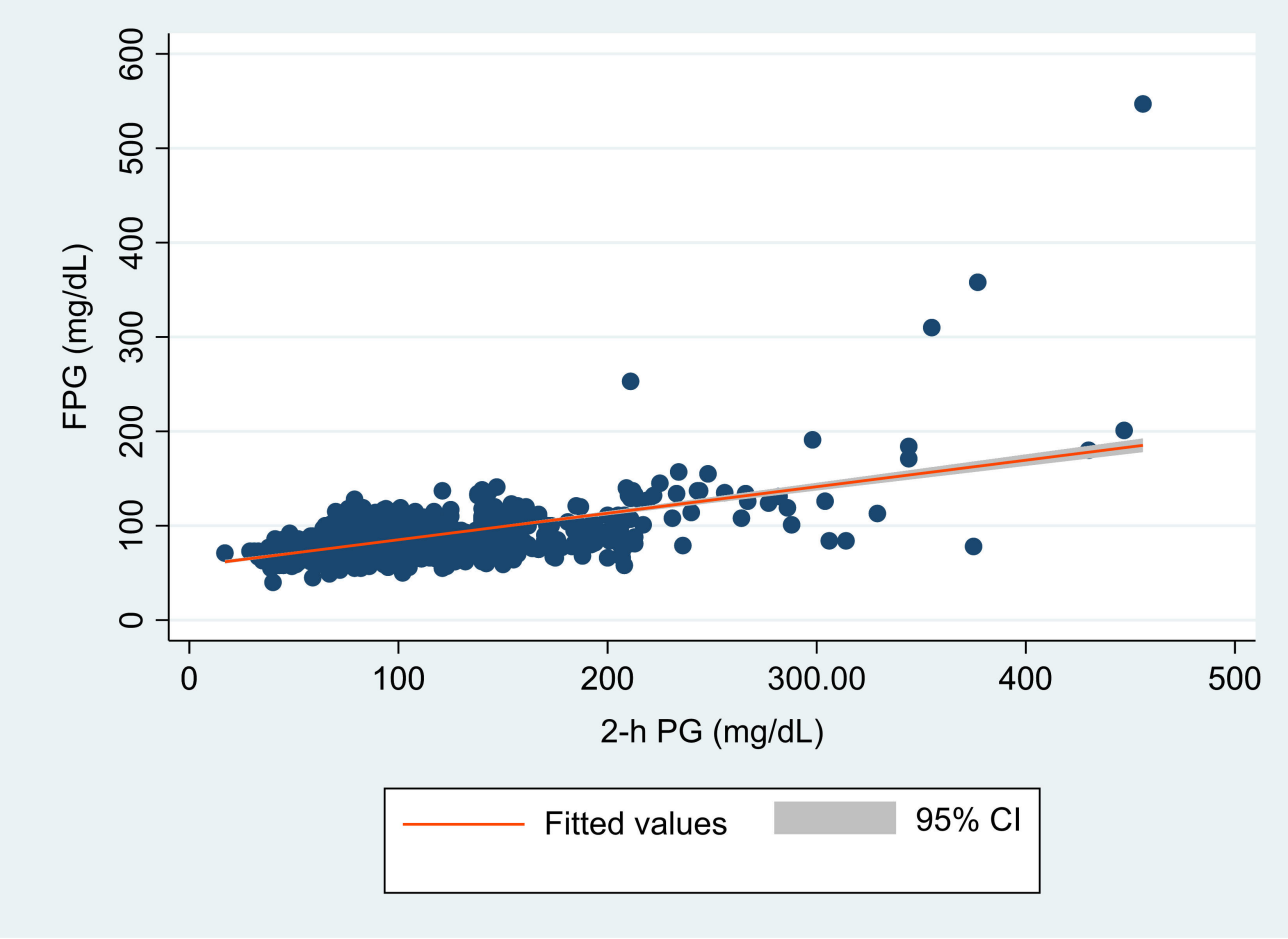
Scatter plot of hour plasma glucose (2-h PG, in mg/dL) and fasting plasma glucose (FPG in mg/dL) with a fitted regression line and 95% confidence interval (CI). The figure demonstrates the overall correlation between FPG and 2-hr PG.

The regression analysis revealed a statistically significant, moderately robust positive correlation (*r*= 0.57, *P* < 0.0001) between FPG and 2-h PG. Employing regression analysis, an equivalent FPG value was derived in relation to 2-h PG (**Figure 2**). Both FPG and 2-h PG demonstrated a strong correlation within a linear relationship represented by FPG (mg/dL) = 57.05 + 0.28 × 2-h PG (mg/dL). Utilizing this equation, an FPG level of 113 mg/dL (6.3 mmol/L) corresponds to a 2-h PG level of 200mg/dL (11.1mmol/L).

The overall concordance between FPG and 2-h PG in identifying diabetes and prediabetes was moderate, with kappa statistic of 0.56 (95% CI, 0.51 – 0.61; p<0.0001) for diabetes and 0.45 (95% CI, 0.40 – 0.50; p<0.0001) for prediabetes. More specifically, the concordance between these two methods for detection of negative cases is very high with a negative predictive value (NPV) of 97.9%.

**Figure 3:**
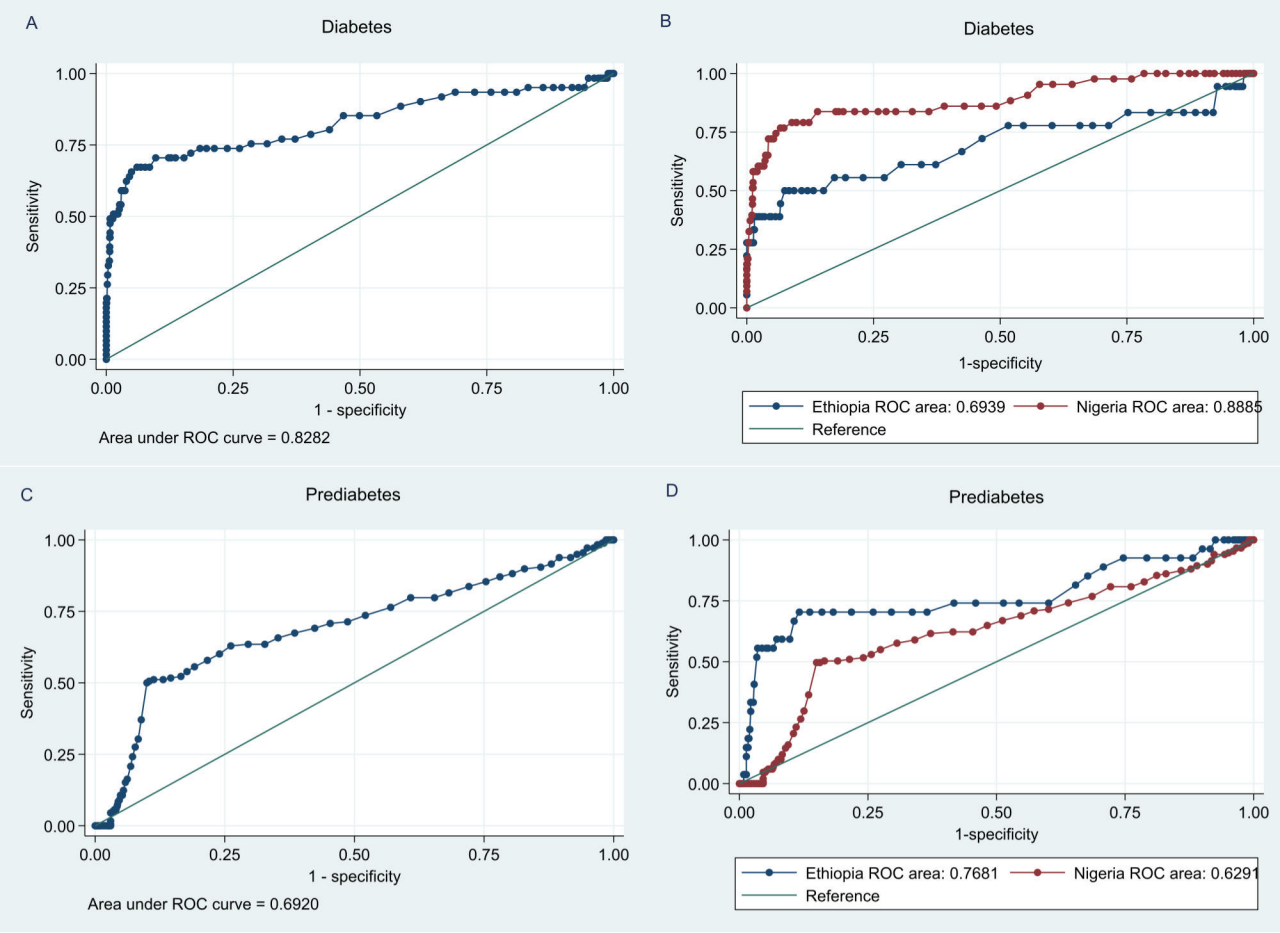
Receiver operating characteristic (ROC) curve for fasting plasma glucose (FPG) to assess the presence of undiagnosed diabetes and prediabetes. These figures show the overall AUC for diabetes **(A)**, the AUC for diabetes in each country **(B)**, overall AUC for prediabetes **(C)** and the AUC for prediabetes in each country **(D)**.

The ROC for each calculated FPG test and cut-off points to identify cases without diabetes and prediabetes is outlined in **Figure 3**. **Table 2**. The FPG test exhibited good and acceptable discriminatory power in identifying individuals with no diabetes, an overall AUC of 0.83 (95% CI: 0.76.-0.90) (**Figure 3A**, **Table 2**). The AUC for men, 0.88 (95% CI: 0.80-0.95), was higher than that for women, 0.77 (95% CI: 0.65-0.89), but this difference was statistically insignificant (P=0.142) (**Table 2**). Moreover, the AUC for Nigerian participants, 0.89 (95% CI: 0.83-0.95) was significantly higher than that of Ethiopian counterparts, 0.70 (95% CI: 0.53-0.86) (P-value = 0.034) (**Figure 3B**, **Table 2**).

**Table 2:** Area under the curve (AUC) for fasting plasma glucose (FPG) to assess the presence of undiagnosed diabetes and prediabetes by country and sex.

Variables	Prediabetes			Diabetes		
	AUC (95% CI)	χ2	P-value	AUC (95% CI)	χ2	P-value
Overall	0.69 (0.67-0.72)	–	–	0.83 (0.76-0.90)	–	–
Country						
Nigeria	0.63 (0.58-0.68)	4.26	0.039	0.89 (0.83-0.95)	4.50	0.034
Ethiopia	0.77 (0.65-0.89)			0.70 (0.53-0.86)		
Sex						
Men	0.68 (0.61-0.74)	0.45	0.505	0.88 (0.80-0.95)	2.09	0.149
Women	0.71 (0.64-0.78)			0.77 (0.64-0.89)		

Diagnostic accuracy of FPG test in distinguishing between prediabetes and diabetes in different groups based on country and sex by usings the area under the curve (AUC) from a Receiver Operating Characteristic (ROC) curve. A higher AUC (closer to 1) indicates better diagnostic performance.

Similarly, the FPG test had good performance in identifying individuals without prediabetes, with an overall AUC of 0.69 (95% CI: 0.67-0.72) (**Figure 3C**, **Table 2**). The performance of the FPG test to identify people without prediabetes revealed a significantly higher AUC among the Ethiopians, 0.77 (95% CI: 0.65-0.89), compared to the Nigerians, 0.63 (95% CI: 0.58-0.68) (**Figure 3D**, **Table 2**). Nonetheless, the performance of the FPG test to identify individuals without prediabetes was comparable between male and female participants (**Table 2**). These ROC curves for FPG were calculated based on the detection of diabetes by 2-h PG ≥ 200 mg/dL and prediabetes by 2-h PG ≥140 mg/dL.

### 3.4 FPG cut-off points for pre-diabetes and diabetes screening

Based on the ROC curve, the optimal cut point of FPG for detecting newly diagnosed diabetes by 2-h PG was determined to be 105 mg/dL, associated with a sensitivity of 67.2% and specificity of 94.0% (AUC 0.83, 95% CI: 0.76 – 0.90; NND 1.6) (**Table 3**). Applying this cut-off point, an additional 59 undiagnosed cases of diabetes (3.8%) among the 1550 participants could be identified.

**Table 3:** Sensitivity and specificity for FPG in diagnosing diabetes and prediabetes.

FPG cut-offs	Diabetes					Prediabetes				
	Se (%)	Sp (%)	Youden J	LR+	LR-	Se (%)	Sp (%)	Youden J	LR+	LR-
80 mg/dL	90.2	38.1	0.282	1.46	0.26	80.4	39.3	0.198	1.326	0.497
85 mg/dL	80.3	56.0	0.363	1.83	0.35	70.1	57.9	0.281	1.667	0.516
90 mg/dL	75.4	71.5	0.469	2.64	0.34	64.1	74.2	0.383	2.487	0.483
95 mg/dL	73.8	81.5	0.553	3.99	0.32	53.8	83.9	0.377	3.338	0.551
100 mg/dL	70.5	87.7	0.582	5.74	0.34	51.6	90.5	0.421	5.421	0.535
105 mg/dL	67.2	94.0	0.612	11.12	0.35	23.4	93.6	0.170	3.667	0.818
110 mg/dL	59.0	96.6	0.557	17.58	0.42	13.6	95.6	0.092	3.091	0.904
115 mg/dL	50.8	97.7	0.485	22.26	0.50	8.7	96.5	0.052	2.473	0.946
120 mg/dL	49.2	98.7	0.479	38.54	0.51	7.6	97.5	0.051	3.055	0.948
125 mg/dL	47.5	99.3	0.468	64.35	0.53	3.3	97.6	0.008	1.349	0.991
130 mg/dL	39.3	99.3	0.387	58.58	0.61	3.3	98.0	0.013	1.649	0.987
135 mg/dL	29.5	99.7	0.292	109.84	0.71	1.6	98.7	0.003	1.236	0.997
140 mg/dL	21.3	99.9	0.212	317.33	0.79	0.5	99.1	-0.003	0.618	1.003
145 mg/dL	19.7	100.0	0.197	Inf	0.80	0.0	99.2	-0.008	0.000	1.008
150 mg/dL	18.0	100.0	0.180	Inf	0.82	0.0	99.3	-0.007	0.000	1.007

Comparison of diagnostic performance metrics for fasting plasma glucose (FPG) cut-off values ranging from 80 mg/dL – 150 mg/dL in diagnosing diabetes and prediabetes to identify which FPG levels offer the best sensitivity, specificity, and diagnostic balance for the two conditions. Youden J is summary statistic combining Se and Sp to show the overall diagnostic effectiveness, it is computed as Youden’s index = Sensitivity + Specificity - 1. A higher value of Youden’s index indicates better overall diagnostic accuracy. FPG, Fasting Plasma Glucose; LR-, negative likelihood ratio; LR+, Positive likelihood ratio; Se, Sensitivity; Sp, Specificity.

Likewise, the optimal cut-off point of FPG in detecting prediabetes diagnosed by 2-h PG was determined to be 99 mg/dL, with a sensitivity of 52.2% and specificity of 90.0% (AUC 0.69, 95% CI:0.67-0.72).

When utilizing the guideline recommended cut-off 126 mg/d, the sensitivity of FPG in identifying diabetes is 44.3%, with a specificity of 99.3%. A lower FPG cut-off value 105 mg/dL revealed markedly increased sensitivity (67.2%) and high specificity (94.0%) for identifying individuals with diabetes. Furthermore, for diagnosing individuals with prediabetes, guideline recommended cut-off value for FPG, 100 mg/dL, demonstrated a sensitivity of 51.6% and specificity of 90.5%. Lowering the FPG cut-off to 90 mg/dL resulted in increased sensitivity (64.1%) and reasonable specificity (74.2%) to identify individuals with pre-diabetes (**Table 3**).

### 3.5 Prediabetes and diabetes missed by FPG and 2-h PG

**Table 4** highlight the percentages of prediabetes and diabetes cases missed by either FPG or 2-h PG alone. Among the total 1550 participants tested by both FPG and 2-h PG, FPG would miss 34.1% of all prediabetes and 44.4% of all diabetes cases, whereas the corresponding percentages missed by 2-h PG were 31.8% and 15.3%, respectively.

**Table 4:** Percentages of diabetes and prediabetes missed by FPG and 2-h PG.

Diabetes							
	All diabetes	Diabetes missed by FPG			Diabetes missed by 2-h PG		
		Missed (%)	Not missed (%)	p-value	Missed (%)	Not missed (%)	p-value
All (N=1550)	72	32 (44.4)	40 (56.6	–	11 (15.3)	60 (84.7)	–
Sex							
Men	43	15 (34.9)	28 (60.1)	0.047	9 (20.9)	34 (79.1)	0.105
Women	29	17 (58.6)	12 (41.4)		2 (6.9)	27 (93.1)	
Age							
< 65 years	62	29 (46.8)	33 (53.2)	0.322	9 (14.5)	53 (85.5)	0.655
≥ 65 years	10	3 (30.0)	7 (70.0)		2 (20.0)	8 (80.0)	
Residence							
Urban	26	12 (46.2)	14 (53.8)	0.826	1 (3.8)	25 (96.2)	0.043
Rural	46	20 (43.5)	26 (56.5)		10 (21.7)	36 (78.3)	
WHO global recommendations on physical activity ^a^							
Optimal	56	24 (42.9)	32 (57.1)	0.612	10 (17.9)	46 (82.1)	0.255
Suboptimal	16	8 (50.0)	8 (50.0)		1 (6.3)	15 (93.7)	
BMI ^b^							
Non-obese	35	21 (60.0)	14 (40.0)	0.013	1 (2.9)	34 (97.1)	0.004
Obese	36	11 (30.6)	25 (69.4)		10 (27.8)	26 (72.2)	
Waist circumference ^c^							
Low risk	31	15 (48.4)	16 (51.6)	0.484	7 (22.6)	24 (77.4)	0.302
Increased risk	16	5 (31.3)	11 (68.8)		2 (12.5)	14 (87.5)	
Substantially increased risk	25	12 (48.0)	13 (52.0)		2 (8.0)	23 (92.0)	
Raised BP							
Yes	33	14 (42.4)	19 (57.6)	0.751	5 (15.2)	28 (84.8)	0.978
No	39	18 (46.2)	21 (53.9)		6 (15.4)	33 (84.6)	
Prediabetes							
	All prediabetes	Prediabetes missed by FPG			Prediabetes missed by 2hPG		
		Missed (%)	Not missed (%)	p-value	Missed (%)	Not missed (%)	p-value
All (N=1550)	261	89 (34.1)	172 (65.9)	–	83 (31.8)	178 (68.2)	–
Sex							
Men	145	52 (35.9)	93 (64.1)	0.502	45 (31.0)	100 (69.0)	0.766
Women	116	37 (31.9)	79 (68.1)		38 (32.8)	78 (67.2)	
Age							
< 65 years	202	60 (29.7)	142 (70.3)	0.006	72 (35.6)	130 (64.4)	0.014
≥ 65 years	59	29 (49.2)	30 (50.8)		11 (18.6)	48 (81.4)	
Country							
Ethiopia	40	13 (32.5)	27 (67.5)	0.817	13 (32.5)	27 (67.5)	0.918
Nigeria	221	76 (34.4)	145 (65.6)		70 (31.7)	151 (68.3)	
Residence							
Urban	125	34 (27.2)	91 (72.8)	0.024	44 (35.2)	81 (64.8)	0.258
Rural	136	55 (40.4)	81 (59.6)		39 (28.7)	97 (71.3)	
WHO global recommendations on physical activity ^a^							
Optimal	212	75 (35.4)	137 (64.6)	0.365	68 (32.1)	144 (67.9)	0.843
Suboptimal	49	14 (28.6)	35 (71.4)		15 (30.6)	34 (69.4)	
BMI ^b^							
Non-obese	168	64 (38.1)	104 (61.9)	0.065	55 (32.7)	113 967.3)	0.525
Obese	90	24 (26.7)	66 (73.3)		26 (8.9)	64 (71.1)	
Waist circumference ^c^							
Normal risk	126	49 (38.9)	77 (61.1)	0.265	42 (33.3)	84 (66.7)	0.594
Increased risk	47	15 (31.9)	32 (68.1)		12 (25.5)	35 (74.5)	
Substantially increased risk	88	25 (28.4)	63 (71.6)		29 (33.0)	59 (67.0)	
Raised BP							
Yes	107	41 (38.3)	66 (61.7)	0.231	33 (30.8)	74 (69.2)	0.781
No	154	48 (31.2)	106 (68.8)		50 (32.5)	104 (67.5)	

a According to WHO global recommendations on physical activity for health optimal physical activity is 150 minutes of moderate-intensity physical activity; or 75 minutes of vigorous-intensity physical activity; or an equivalent combination of moderate- and vigorous-intensity physical activity achieving at least 600 MET-minutes.

b obese is based on WHO BMI classification (BMI ≥ 30 kg/m^2^).

c Waist circumference classification is based on WHO recommendation where normal risk is ≤94 cm for men and ≤80 cm for women; increased risk is >94 – 102 cm for men and >80 – 88 cm for women; and substantial increased risk is >102 cm for men and >88 cm for women.

BP, blood pressure; FPG, fasting plasma glucose; 2-h PG, 2-hour plasma glucose; BMI, body mass index.

The rates of missed prediabetes cases using FPG and 2-h PG were similar for men and women study participants. In contrast, FPG demonstrated a higher rate of missed cases of diabetes in women compared to men (p = 0.047), whereas 2-h PG presented a higher rate of missed diabetes cases in men compared to women, despite this was not statistically significant.

Moreover, the rate of missed cases of prediabetes by FPG was higher among individuals with aged 70 years and above as compared to middle-aged and young study participants (p = 0.006). The percentage of missed diabetes cases by FPG was significantly higher among non-obese participants compared to obese individuals with diabetes (p = 0.013), while the opposite trend was observed in 2-h PG. However, there was no significant difference in the rate of missed prediabetes cases by FPG and 2-h PG among obese and non-obese individuals.

## 4 Discussion

The performance of fasting plasma glucose (FPG) to identify undiagnosed prediabetes and diabetes cases has been investigated based on 2-hour plasma glucose (2-h PG) during an oral glucose tolerance test (OGTT)in this study. According to ADA and IDF, both FPG and 2-h PG during 75-g OGTT are equally appropriate for screening of diabetes (4, 8), but the agreement between them was moderate for identifying diabetes and prediabetes, indicating that these tests did not consistently categorize the same individuals with abnormal blood glucose. Existing evidence from western populations and this study suggests limited agreement between FPG and 2-h PG. In the National Health and Nutrition Examination Survey (NHANES) in the US, concordance between FPG and 2-h PG was limited with a kappa statistic of 0.310 (24). Nevertheless, the concordance of FPG with 2-h PG to identify non-diabetes individuals is very high with a negative predictive value (NPV) of 97.9%. In previous studies, the agreement between FPG and 2-h PG was shown to be inconsistent, implicating the call for comprehensive studies across diverse populations to improve guidelines for the diagnosis of diabetes.

Most importantly, with the current study FPG was able to correctly identify 99.3% of individuals with no diabetes as measured by the 2-h PG. Several community-based studies also reported FBP is highly specific to rule-out diabetes. In an Iranian study from diabetes screening program, the specificity of FBG was comparable to the current study reported at 95.7%, indicating its reliability in screening in the community (25). The DECODE study compared the diagnostic performance of FPG and 2-h PG in large European populations. The study found that FPG had high specificity for identifying individuals without diabetes, with a low rate of misclassification when compared to the 2-h PG (26). The ADA guidelines indicated that FPG is a reliable screening method for identifying individuals without diabetes (4). Hence, results of the current and previous studies indicate that the usefulness of FPG for screening of diabetes and prediabetes in this and other populations.

Although FPG is reported to have higher specificity and lower intra-individual coefficients of variation compared to 2-h PG (27), it was found to identify a smaller number of individuals with diabetes (28, 29). This study showed that a significant number of individuals with diabetes would have been missed if the screening relied only on FPG. Moreover, while about three-fourth (72.5%) of individuals screened to be diabetes through FPG were also detected by 2-h PG, the latter disclosed an additional 2.1% prevalence of diabetes. These findings were consistent with results from earlier studies (30–32). In addition, studies in Africa have consistently reported that the use of FPG alone identified fewer diabetes cases and IGT compared to 2-h PG (33, 34). The percentage of individuals missed by FPG in our study was similar to findings from Cape Town, South Africa, where about 44% of those with OGTT-detected diabetes missed (34). But this percentage was higher than the DECODE report (30%) in Europe (35) and much lower than the percentage recorded in France, where 71% of the diabetes diagnoses would have gone undetected if the measurement was FPG alone (30). Moreover, as indicated in the report of WHO and IDF meeting, false negative and false positive results are inevitable given that any initial screening test is not a full diagnostic test (36). The study’s findings emphasize the need for complementary tests, such as 2-h PG, to mitigate the limitations of a single screening method. By using multiple tests, healthcare providers can reduce the likelihood of missing true cases (false negatives) and improve the overall reliability of diabetes diagnosis.

As recommended by the 2019 European Society of Cardiology (ESC) guidelines on diabetes, pre-diabetes, and CVDs developed in collaboration with the European Association for the Study of Diabetes (EASD), it may be wise to implement a sequential screening framework (17). In this strategy, the FPG test serves as the initial blood glucose test. An FPG ≥105% is diagnostic for diabetes, whereas an additional 2-h PG test is recommended in individuals who did not fulfil FPG criteria but can be considered at high risk of developing diabetes. When this sequential approach would have been used in this population, about two-third of the diabetes cases would have been identified by FPG alone. Screening in this manner is less laborious with a reasonable cost and thus might be a better patient care practice by prioritizing those individuals with a genuine need for 2-h PG, thereby mitigating the risk of overdiagnosis.

Screening in an apparently healthy population with lower FPG cut-off point may enhance the early detection of individuals with IGT. The current study has determined the optimal FPG cut-off values for identifying individuals with prediabetes and diabetes. This emphasizes the need to reassess screening thresholds for identifying diabetes and prediabetes in the African populations, given their significant importance for public health. The FPG cut-off value of 126 mg/dL had a very high specificity (99.3%) for identifying diabetes cases but had a relatively lower sensitivity (47.5%). Variations in sensitivity ranging from 40% to 94% and specificity from 83% to 100% for FPG 126 mg/dL was reported in a systematic review and meta-analysis (10). Once the cut-off value was lowered furthermore it improved the sensitivity at the expense of a minor lowering of specificity. We identified an FPG level of 99 mg/dL and 105 mg/dL as the optimal cut-off values for our population in screening pre-diabetes and diabetes cases, respectively. Our finding of the optimal cut-off for FPG is consistent with the 104 mg/dL cut-off reported in a systematic review and meta-analysis for detecting diabetes (10). But our finding of optimal threshold for FPG differs from that estimated by Hoyer, et al., 2018 (37).

Studies from Asian countries have also suggested reduced threshold values for FPG in the range of 95.0–113.4 mg/dL for screening diabetes (38–40). Similar to our results for screening pre-diabetes, other studies recommended a threshold value of 100 mg/dL as a good cut-off with acceptable sensitivity and specificity (13, 40).

In this research, FPG showed a higher rate of missing diabetes cases in women compared to men. This was in line with the NHANES study in the US where there were more women with isolated 2-h PG ≥200 mg/dL, while there were more men with isolated FPG ≥126 mg/dL (24). A study by Kim et al. found that FPG had lower sensitivity for detecting diabetes in women, particularly those with a history of gestational diabetes mellitus (GDM), who often have high level of postprandial plasma glucose rather than elevated FPG levels (41). Previous researches have also reported that women have lower FPG and higher 2-h PG levels (42–44). This confirms that the SSA women face a higher risk of developing diabetes following GDM, which often goes undetected due to scares resources for screening. The combination of undetected GDM and the lower sensitivity of FPG in women creates a significant public health challenge for SSA women. Without effective screening and diagnostic tools, many women at risk of T2DM remain undiagnosed until complications arise (4).

While FPG exhibited a peak in the 45–59 age group, 2-h PG demonstrated a linear increase with advancing age. Likewise, research in other countries have also demonstrated a significant increase in both FPG and 2-h PG after OGTT with aging, as highlighted by Chia (45) and Kalyani (46). In the present study, the 2-h PG criterion was better in identifying older adult individuals with IGT than younger participants as older individuals (≥70 yrs) had higher percentages of missed IFG than the middle-aged and young individuals. The NHANES survey also reported that diabetes cases with isolated 2-h PG ≥200 mg/dL were older than the diabetes cases with isolated FPG ≥126 mg/dL (24). This may partly attribute to decreased muscle mass and reduced physical activity with aging. This underscores the significance of including post-OGTT PG levels to enhance sensitivity in identifying diabetes among older adult individuals in our population.

It is necessary to keep in mind that the importance of an early screening of IFG or IGT lies in its ability to predict diabetes in the future. In this context, while reducing the IFG threshold to 100 mg/dL enhances the predictive capacity of IFG for identifying IGT, it concurrently increases the number of false positive cases. This finding has triggered discussions on the public health merits of lowering the threshold for normal FPG (47, 48). It can be established that with an increasing threshold, the sensitivity of FPG test decreases while the specificity increases. In cases where a higher level of sensitivity is required, such as in screening for type 2 diabetes, a lower threshold may be preferable. It is important to note that establishing a diagnostic threshold for screening involves considerations of cost-effectiveness. Therefore, setting a screening threshold should not solely rely on estimated sensitivities and specificities; clinical and health-economic parameters shall also be taken into account. On the other hand, if the intention is to utilize highly specific tests for confirming a diagnosis, then higher thresholds should be considered.

The European Society of Cardiology (ESC) guidelines emphasized that while screening for diabetes is critical, it alone does not address the broader public health challenges in resource-limited settings such as SSA (17). Instead, it should be targeting cost-effective and accessible screening methods, even if they are invasive, to ensure accurate identification of at-risk individuals. More accurate diagnostic methods are in demand for individuals who do not test negative, ensuring that resources are used efficiently to tackle the growing burden of diabetes and its complications. Therefore, clinicians need to consider moving beyond reliance on Western guidelines and advocate for locally tailored screening guidelines. Specifically, practitioners in SSA are recommended to develop locally validated FPG thresholds and confirm with subsequent diagnostic tests like 2-h PG or HbA1c, particularly for individuals at high-risk or with borderline test results. Women, who are more likely to be missed by FPG, should be screened more cautiously, with additional tests considered if clinical suspicion remains significant. By adapting practices to the local context, SSA can improve diabetes detection and reduce underdiagnosis, addressing the region’s unique challenges more effectively.

## 5 Strength and limitation

The study employed a community-based, multi-center design on 1550 study participants from Sub-Sahara Africa. The diagnostic capacity of FPG was evaluated against the reference 2-h PG test, to our knowledge, one of the first such studies in the SSA population. Furthermore, setting the FPG cut-off level corresponding to a 2-h PG of 200 mg/dL, the gold standard for diabetes diagnosis, substantially adds to the novelty of this study in the SSA setting. Nevertheless, a single FPG or 2-h PG measurement was utilized to identify individuals with pre-diabetes or diabetes while the criteria for the diagnosis of diabetes requires repeated measurement in asymptomatic individuals. However, given the large study population, the consequences of this limitation are expected to be negligible.

## 6 Conclusion

FPG was able to correctly identify 99.3% of individuals with no diabetes indicating its usefulness for screening of diabetes, But, a significant percentage (44.4%) of diabetes cases would have remained undetected if only FPG had been utilized instead of the 2-h PG. The use of a lower FPG cut-off value at 105 mg/dL enhanced the detection of individuals with diabetes. Likewise, an FPG cut-off at 99 mg/d, comparable to that of ADA and IDF, can improve early detection of individuals at risk of type 2 diabetes. The use of 2-h PG test is recommended to identify older individuals, females and non-obese persons with diabetes who would be missed by measuring only FPG. In all cases implementing a sequential screening model has the potential to enhance patient care, minimize unnecessary over-diagnosis, and optimize efficient resource use.

Overall, re-evaluation of screening thresholds for FPG for the early identification of individuals with diabetes and prediabetes has pivotal public health implications. Our study emphasizes the need for a thorough and careful revision of diabetes screening guidelines tailored to demographic, clinical, and lifestyle factors influencing the screening and diagnosis of diabetes.

## Data Availability

The original contributions presented in the study are included in the article/supplementary material. Further inquiries can be directed to the corresponding author/s.

## References

[1] Zhou B Rayner AW Gregg EW Sheffer KE Carrillo-Larco RM Bennett JE. Worldwide trends in diabetes prevalence and treatment from 1990 to 2022: a pooled analysis of 1108 population-representative studies with 141 million participants. Lancet. (2024) 404:2077–93. doi: 10.1016/S0140-6736(24)02317-1 PMC7616842 39549716

[2] Sun H Saeedi P Karuranga S Pinkepank M Ogurtsova K Duncan BB. IDF Diabetes Atlas: Global, regional and country-level diabetes prevalence estimates for 2021 and projections for 2045. Diabetes Res Clin practice. (2022) 183:109119. doi: 10.1016/j.diabres.2021.109119 PMC11057359 34879977

[3] Ogurtsova K Guariguata L Barengo NC Ruiz PL-D Sacre JW Karuranga S. IDF diabetes Atlas: Global estimates of undiagnosed diabetes in adults for 2021. Diabetes Res Clin practice. (2022) 183:109118. doi: 10.1016/j.diabres.2021.109118 34883189

[4] American Diabetes Association Professional Practice Committee. 2. Classification and diagnosis of diabetes: Standards of Medical Care in Diabetes—2022. Diabetes Care. (2022) 45:S17–38. doi: 10.2337/dc22-S002 34964875

[5] Aschner P. New IDF clinical practice recommendations for managing type 2 diabetes in primary care. Diabetes Res Clin practice. (2017) 132:169–70. doi: 10.1016/j.diabres.2017.09.002 28962686

[6] Davidson KW Barry MJ Mangione CM Cabana M Caughey AB Davis EM. Screening for prediabetes and type 2 diabetes: US Preventive Services Task Force recommendation statement. Jama. (2021) 326:736–43. doi: 10.1001/jama.2021.12531 34427594

[7] Khunti K Davies M. Should we screen for type 2 diabetes: Yes. Bmj. (2012) 345:e4514. doi: 10.1136/bmj.e4514 22777029

[8] Aschner P Karuranga S James S Simmons D Basit A Shaw JE. The International Diabetes Federation’s guide for diabetes epidemiological studies. Diabetes Res Clin Pract. (2021) 172. doi: 10.1016/j.diabres.2020.108630 33347900

[9] Saudek CD Kalyani RR Derr RL. Assessment of glycemia in diabetes mellitus: hemoglobin A1c. J Assoc Physicians India. (2005) 53:299–305. 15987016

[10] Kaur G Lakshmi PVM Rastogi A Bhansali A Jain S Teerawattananon Y. Diagnostic accuracy of tests for type 2 diabetes and prediabetes: A systematic review and meta-analysis. PloS One. (2020) 15:e0242415. doi: 10.1371/journal.pone.0242415 33216783 PMC7678987

[11] Droumaguet C Balkau B Simon D Caces E Tichet J Charles MA. Use of HbA1c in predicting progression to diabetes in French men and women: data from an Epidemiological Study on the Insulin Resistance Syndrome (DESIR). Diabetes Care. (2006) 29:1619–25. doi: 10.2337/dc05-2525 16801588

[12] Ryu S Shin H Chang Y Sung KC Song J Lee SJ. Should the lower limit of impaired fasting glucose be reduced from 110 mg/dL in Korea? Metabolism. (2006) 55:489–93. doi: 10.1016/j.metabol.2005.10.010 16546479

[13] Inoue K Matsumoto M Akimoto K. The threshold for definition of impaired fasting glucose in a Japanese population. Diabetes Med. (2009) 26:1175–8. doi: 10.1111/j.1464-5491.2009.02850.x 19929998

[14] Kato M Noda M Suga H Matsumoto M Kanazawa Y. Fasting plasma glucose and incidence of diabetes — implication for the threshold for impaired fasting glucose: results from the population-based Omiya MA cohort study. J Atheroscler Thromb. (2009) 16:857–61. doi: 10.5551/jat.1792 20032586

[15] Noda M Kato M Takahashi Y Matsushita Y Mizoue T Inoue M. Fasting plasma glucose and 5-year incidence of diabetes in the JPHC diabetes study - suggestion for the threshold for impaired fasting glucose among Japanese. Endocr J. (2010) 57:629–37. doi: 10.1507/endocrj.K10E-010 20508383

[16] American Diabetes Association. Diagnosis and classification of diabetes mellitus. Diabetes Care. (2013) 37:S81–90. doi: 10.2337/dc14-S081 24357215

[17] Cosentino F Grant PJ Aboyans V Bailey CJ Ceriello A Delgado V. 2019 ESC Guidelines on diabetes, pre-diabetes, and cardiovascular diseases developed in collaboration with the EASD. Eur Heart J. (2020) 41:255–323. doi: 10.1093/eurheartj/ehz486 31497854

[18] International Diabetes Federation. IDF diabetes atlas. 10th edn. Brussels, Belgium: International Diabetes Federation (2021). Available at: https://www.diabetesatlas.org (Accessed November 10, 2022).

[19] WHO Consultation on Obesity. Obesity: preventing and managing the global epidemic Vol. 894. Geneva, Switzerland: World Health Organization technical report series (2000) p. 1–253. 11234459

[20] World Health Organization. Waist circumference and waist-hip ratio: report of a WHO expert consultation Vol. 64. Geneva, Switzerland: World Heal Organ (2008) p. 8–11.

[21] World Health Organization. Global physical activity questionnaire (GPAQ) analysis guide. Geneva: World Health Organization (2012).

[22] R Core Team. R: A Language and environment for statistical computing. (2023). (Vienna, Austria: R foundation for statistical computing).

[23] Nahm FS. Receiver operating characteristic curve: overview and practical use for clinicians. Korean J Anesthesiol. (2022) 75:25–36. doi: 10.4097/kja.21209 35124947 PMC8831439

[24] Huang J Ou HY Karnchanasorn R Samoa R Chuang LM Chiu KC. Clinical implication of fasting and post-challenged plasma glucose in diagnosis of diabetes mellitus. Endocrine. (2015) 48:511–8. doi: 10.1007/s12020-014-0301-3 24895042

[25] Kianpour F Fararouei M Hassanzadeh J Mohammadi M Dianatinasab M. Performance of diabetes screening tests: an evaluation study of Iranian diabetes screening program. Diabetol Metab Syndr. (2021) 13:13. doi: 10.1186/s13098-021-00632-9 33499908 PMC7836149

[26] Group DS Group obotEDE. Glucose tolerance and mortality: comparison of WHO and American Diabetes Association diagnostic criteria. The DECODE study group. European Diabetes Epidemiology Group. Diabetes Epidemiology: Collaborative analysis Of Diagnostic criteria in Europe. Lancet. (1999) 354:617–21. doi: 10.1016/S0140-6736(98)12131-1 10466661

[27] Barr RG Nathan DM Meigs JB Singer DE. Tests of glycemia for the diagnosis of type 2 diabetes mellitus. Ann Intern Med. (2002) 137:263–72. doi: 10.7326/0003-4819-137-4-200208200-00011 12186517

[28] Sinnott M Kinsley BT Jackson AD Walsh C O’Grady T Nolan JJ. Fasting plasma glucose as initial screening for diabetes and prediabetes in irish adults: The Diabetes Mellitus and Vascular health initiative (DMVhi). PloS One. (2015) 10:e0122704. doi: 10.1371/journal.pone.0122704 25874867 PMC4398404

[29] Aekplakorn W Tantayotai V Numsangkul S Sripho W Tatsato N Burapasiriwat T. Detecting prediabetes and diabetes: agreement between fasting plasma glucose and oral glucose tolerance test in Thai adults. J Diabetes Res. (2015) 2015:396505. doi: 10.1155/2015/396505 26347060 PMC4543794

[30] Cosson E Hamo-Tchatchouang E Banu I Nguyen MT Chiheb S Ba H. A large proportion of prediabetes and diabetes goes undiagnosed when only fasting plasma glucose and/or HbA1c are measured in overweight or obese patients. Diabetes Metab. (2010) 36:312–8. doi: 10.1016/j.diabet.2010.02.004 20627649

[31] Alberti KG Zimmet P Shaw J. International Diabetes Federation: a consensus on Type 2 diabetes prevention. Diabetes Med. (2007) 24:451–63. doi: 10.1111/j.1464-5491.2007.02157.x 17470191

[32] Cowie CC Rust KF Ford ES Eberhardt MS Byrd-Holt DD Li C. Full accounting of diabetes and pre-diabetes in the U.S. population in 1988-1994 and 2005-2006. Diabetes Care. (2009) 32:287–94. doi: 10.2337/dc08-1296 PMC2628695 19017771

[33] Werfalli M Engel ME Musekiwa A Kengne AP Levitt NS. The prevalence of type 2 diabetes among older people in Africa: a systematic review. Lancet Diabetes Endocrinol. (2016) 4:72–84. doi: 10.1016/S2213-8587(15)00363-0 26548379

[34] Kengne AP Erasmus RT Levitt NS Matsha TE. Alternative indices of glucose homeostasis as biochemical diagnostic tests for abnormal glucose tolerance in an African setting. Prim Care Diabetes. (2017) 11:119–31. doi: 10.1016/j.pcd.2017.01.004 28132763

[35] Borch-Johnsen K Tuomilehto J Balkau B Qiao Q. Is fasting glucose sufficient to define diabetes? Epidemiological data from 20 European studies. The DECODE-study group. European Diabetes Epidemiology Group. Diabetes Epidemiology: Collaborative analysis of Diagnostic Criteria in Europe. Diabetologia. (1999) 42:647–54. doi: 10.1007/s001250051211 10382583

[36] World Health Organization Chronic Respiratory and Diseases Arthritis Team. Screening for type 2 diabetes: report of a World Health Organization and International Diabetes Federation meeting. (Geneva, Switzerland: World Health Organization) (2003).

[37] Hoyer A Rathmann W Kuss O. Utility of HbA(1c) and fasting plasma glucose for screening of Type 2 diabetes: a meta-analysis of full ROC curves. Diabetes Med. (2018) 35:317–22. doi: 10.1111/dme.2018.35.issue-3 29230866

[38] Doi Y Kubo M Yonemoto K Ninomiya T Iwase M Arima H. Fasting plasma glucose cutoff for diagnosis of diabetes in a Japanese population. J Clin Endocrinol Metab. (2008) 93:3425–9. doi: 10.1210/jc.2007-2819 18559920

[39] Mukai N Doi Y Ninomiya T Hata J Hirakawa Y Fukuhara M. Cut-off values of fasting and post-load plasma glucose and HbA1c for predicting Type 2 diabetes in community-dwelling Japanese subjects: the Hisayama Study. Diabetes Med. (2012) 29:99–106. doi: 10.1111/j.1464-5491.2011.03378.x 21726278

[40] Katulanda GW Katulanda P Dematapitiya C Dissanayake HA Wijeratne S Sheriff MHR. Plasma glucose in screening for diabetes and pre-diabetes: how much is too much? Analysis of fasting plasma glucose and oral glucose tolerance test in Sri Lankans. BMC Endocrine Disord. (2019) 19:11. doi: 10.1186/s12902-019-0343-x PMC6341544 30670002

[41] Kim C Newton KM Knopp RH. Gestational diabetes and the incidence of type 2 diabetes: a systematic review. Diabetes Care. (2002) 25:1862–8. doi: 10.2337/diacare.25.10.1862 12351492

[42] Sicree RA Zimmet PZ Dunstan DW Cameron AJ Welborn TA Shaw JE. Differences in height explain gender differences in the response to the oral glucose tolerance test- the AusDiab study. Diabetes Med. (2008) 25:296–302. doi: 10.1111/j.1464-5491.2007.02362.x 18307457

[43] Williams JW Zimmet PZ Shaw JE de Courten MP Cameron AJ Chitson P. Gender differences in the prevalence of impaired fasting glycaemia and impaired glucose tolerance in Mauritius. Does sex matter? Diabetes Med. (2003) 20:915–20. doi: 10.1046/j.1464-5491.2003.01059.x 14632717

[44] Tripathy D Carlsson M Almgren P Isomaa B Taskinen MR Tuomi T. Insulin secretion and insulin sensitivity in relation to glucose tolerance: lessons from the Botnia Study. Diabetes. (2000) 49:975–80. doi: 10.2337/diabetes.49.6.975 10866050

[45] Chia CW Egan JM Ferrucci L. Age-related changes in glucose metabolism, hyperglycemia, and cardiovascular risk. Circ Res. (2018) 123:886–904. doi: 10.1161/CIRCRESAHA.118.312806 30355075 PMC6205735

[46] Kalyani RR Egan JM. Diabetes and altered glucose metabolism with aging. Endocrinol Metab Clin North Am. (2013) 42:333–47. doi: 10.1016/j.ecl.2013.02.010 PMC3664017 23702405

[47] Davidson MB Landsman PB Alexander CM. Lowering the criterion for impaired fasting glucose will not provide clinical benefit. Diabetes Care. (2003) 26:3329–30. doi: 10.2337/diacare.26.12.3329 14633823

[48] Genuth S. Lowering the criterion for impaired fasting glucose is in order. Diabetes Care. (2003) 26:3331–2. doi: 10.2337/diacare.26.12.3331 14633824

